# Vascular plant and bryophyte species richness in response to water quality in lowland spring niches with different anthropogenic impacts

**DOI:** 10.1007/s10661-018-6703-6

**Published:** 2018-05-10

**Authors:** Katarzyna Puczko, Piotr Zieliński, Szymon Jusik, Anita Kołakowska, Elżbieta Jekatierynczuk-Rudczyk

**Affiliations:** 1 Department of Environmental Protection, Institute of Biology, University of Bialystok, Ciołkowskiego 1J, 15-245 Białystok, Poland; 2 Department of Ecology and Environmental Protection, Poznan University of Life Sciences, Wojska Polskiego 28, 60-637 Poznań, Poland

**Keywords:** Springs, Plants, Plant biodiversity, Crenophytes, Water chemistry

## Abstract

Many freshwater ecosystems face severe threats from anthropogenic disturbances. In the field, we investigated the morphology of spring niches and the species richness of vascular plants and bryophytes in 16 springs, draining the quaternary aquifers, located in two different environments—an urban area (city of Białystok) and a protected area (Knyszyn Forest Landscape Park, NE Poland). In total, 47 vascular plant and 45 bryophyte species were recorded, representing mostly crenophytes including protected species. The most important water quality parameters that can be used to evaluate variations of the spring water chemistry in NE Poland are the mineral-related parameters (electrolytic conductivity, Ca^2+^, SO_4_^2−^, and Cl^−^). The organic-related parameters (DOC) and nutrients (TP, NO_3_^−^-N) were negatively involved in water quality variations. Our results show that anthropogenic activity significantly affects the biodiversity of plant communities in lowland springs. The presence or absence of crenophytes and bryophytes is indicative of the ecological status of the groundwater outflow complexes.

## Introduction

Natural outflows of groundwater are complex ecosystems in both hydrological and biotic terms. They are characterized, in most cases, by a high biodiversity of flora and fauna (Springer and Stevens 2009; Warncke 1980). Springs occur more frequently in mountains and upland areas, and there are fewer reports on the biology and ecology of typical springs in lowlands of Central Europe (Chełmicki et al. 2011; Osadowski and Strzelczak 2010). Further, their presence in urban areas is very rare (Jekatierynczuk-Rudczyk 2008).

Erosion activity by spring waters causes a spring niche to develop. Springs have been considered to be stabile habitats that harbor stable communities (Juutinen 2011). Spring niches such as algae habitats indicate the species richness of diatoms (Żelazna-Wieczorek and Maninska 2006; Wojtal 2013). Springs are inhabited by highly specialized plants that are highly tolerant of low water temperature, so-called crenophytes. Natural outflows of groundwater are an important element of a hydrographic network shaping water relations in a particular area and directly affecting the water balance of a catchment. Within the spring niche, the energy exchange processes at the soil-water-air interface mean that in temperate climates, the outflows never freeze (Czarnecka and Janiec 2007).

Plant communities of springs differ from other plant communities by their dependence on permanent, relatively cold water abundant in oxygen. In the spring niches where plant cover is developed, water cannot easily flow away, and the soil water reserve can thus be enriched (Hadač 1983). Differentiation of environmental factors affects the abundance and distribution of plants associated with outflows of groundwater. The decisive factors are those directly related to the outflow, such as geological structure, types of sediments, topography, climate, and hydrology, which affect the water quality (Lacoul and Freedman 2006; Michalik 2008). Habitat conditions (primarily in terms of the substrate) within the niche are largely mosaic, and this is reflected in their floristic diversity. Mid-forest springs are characterized by particular floristic abundance, containing abundant bryophytes and vascular plants from different habitats: aquatic, peat, reed, and forest species (Kucharski 2007).

Urban flora and vegetation have specific features and differ significantly from those of surrounding non-urbanized areas (McKinney 2008). Urban infrastructure replaces open land and vegetation, and surfaces that were permeable and moist generally become impermeable and dry (Rysiak and Czarnecka 2015). The most consistent and pervasive effect of urbanization is an increase in an impervious surface cover within urban catchments, altering the hydrology and geomorphology of streams and springs.

The relationship between spring location and environmental characteristics in lowlands has not been studied extensively. The aim of this paper is to compare the vegetation of lowland spring niches in areas with varying degrees of anthropogenic transformation, as well as to study the impact of water quality on the phytocoenosis of springs. We seek to determine what would form the main habitat gradient that could affect community composition for both angiosperms and mosses. We hypothesized that spring hydrochemistry affects bryophytes more strongly than vascular plants.

## Materials and methods

### Study area

**Fig. 1:**
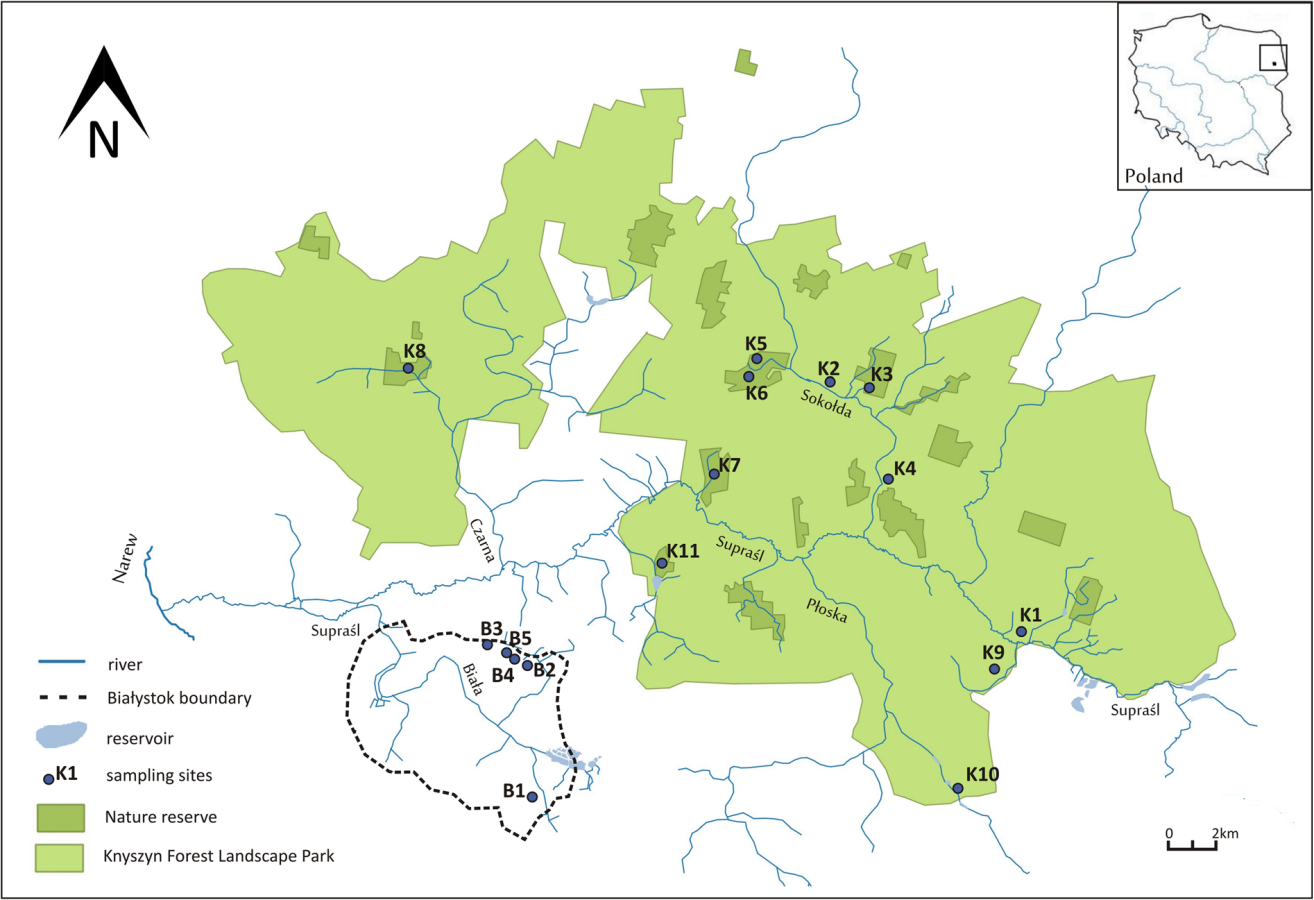
The distribution of study sites in northeastern Poland. Springs in Białystok city are indicated with B1-B5, springs in KFLP are indicated with K1-K11. Coordinates presented along the edges are in ETRS89/Poland CS92

The study was conducted in northeastern Poland, in the area of Knyszyn Forest Landscape Park (KFLP) and in the city of Białystok (Fig. 1). This area is distinguished by a complex hydrographic network that owes its shape to varied terrain formed during the Weichselian glaciation. The area is characterized by the presence of moraine plateaus, the slopes of which are cut by numerous valleys. The outflows of groundwater in the study area are usually located on the slopes of valleys and occasionally in the valley bottoms and riverbeds (Jekatierynczuk-Rudczyk et al. 2017; Jonczak 2013).

KFLP is one of the best preserved forest complexes in Poland that contains varied postglacial sculpture, typical forest streams, and springs. The surface waters in KFLP are almost entirely located in the catchment of Supraśl river (Fig. 1). In KFLP, most of the natural outflows of groundwater are the valley type. The yield of the forest springs varies from 0.02 to 27 dm^3^ s^−1^. However, the most common yield for the forest springs is 0.1 to 0.5 dm^3^ s^−1^ (Jekatierynczuk-Rudczyk 2010).

Białystok, a city with a population of over 280,000 residents, lies in the immediate vicinity of the forest (Fig. 1). The area of the city is mostly deforested, and hydrological conditions are significantly changed. The geological and geomorphological structures are the same types as in the area of KFLP. The area is built from postglacial formations developed during the areal deglaciation of the Weichselian ice sheet. Despite the ongoing horizontal and vertical transformations of surface and groundwaters, the Białystok region still has a well-preserved natural environment, which is confirmed by the presence of spring niches (Jekatierynczuk-Rudczyk 2008). The springs are located in partly forested areas, with the exception of one limnocrene located in a housing estate. The average yields of the investigated urban outflows range from 0.1 to 5.0 dm^3^ s^−1^. Bialystok springs are more exposed to anthropogenic contaminations than are KFLP springs due to leaks in the sewerage network and to surface contamination in infiltration zones and from local landfills. The water quality in urban springs is also endangered by the dense transportation network and the progressive development of the city.

### Methods

The study included 11 forested spring niches located in the area of KFLP and 5 urban spring niches in the city of Białystok (Fig. 1, Table 1). The surface area and saturation zone structure of the spring niches were specified directly in the field. In each of the studied niches, the following three zones can be distinguished: exfiltration of groundwater in the footslope zones, the hyporheic zone covering niche bottoms, and the stream flowing out of the channel head. The detailed study included the flora occupying the hyporheic zone/watered part of the spring niche. The outer boundaries of each spring were delineated to the edge of the niche slope. Studies have not been conducted in the outflow zone, where habitat conditions were considerably altered due to increased flow rate. A land use indicator was developed based on the CORINE land cover data set.

**Table 1:** Morphological characteristics of spring niches in the Supraśl basin (*Q* average [dm^3^ s^−1^]: Jekatierynczuk-Rudczyk 2007, 2008, 2010)

	Geographical coordinates	Hydrological location	*Q* av. [dm^3^ s^−1^]	Type of spring	Form of the niche	Surface of the niche [m^2^]	Geology of the vadose zone	Land use	Anthropogenic impact
B1	53° 05′ 49.3″ N 23° 11′ 59.4″ E	Biała catchment	0.5	Rheocrene	Round	28	Sand, gravel, peat	Forest	High
B2	53° 10′ 39.7″ N 23° 10′ 59.1″ E	Biała catchment	0.3	Rheocrene	Round	500	Sand, gravel, peat	Forest	High
B3	53° 10′ 02.8″ N 23° 11′ 49.7″ E	Jaroszówka catchment	2.4	Artificial limnocrene	Round	450	Sand, peat	Urban area	High
B4	53° 10′ 38.8″ N 23° 11′ 59.1″ E	Jaroszówka catchment	5.2	Rheocrene	Elongated/forked	250	Sand, gravel	Forest	High
B5	53° 10′ 23.9″ N 23° 11′ 52.1″ E	Jaroszówka catchment	0.3	Rheocrene	Elongated	125	Sand, gravel	Forest/grassland	High
K1	53° 09′ 36.8″ N 23° 34′ 26.9″ E	Radulinka catchment	0.5	Rheocrene	Round	312	Peat	Grassland	Low
K2	53° 14′ 39.2″ N 23° 27′ 45.3″ E	Sokołda catchment	n.a.	Rheocrene	Elongated	268	Sand, gravel, peat	Grassland/alder forest	Low
K3	53° 14′ 24.3″ N 23° 27′ 48.9″ E	Sokołda catchment	n.a.	Rheocrene	Elongated/forked	160	Sand, gravel	Grassland/alder forest	Low
K4	53° 13′ 42.3″ N 23° 28′ 19.9″ E	Sokołda catchment	9.9	Limnocrene	Round	180	Sand, gravel	Forest	Low
K5	53° 16′ 52.7″ N 23° 22′ 26.7″ E	Migówka catchment → Sokołda catchment	n.a.	Rheocrene	Round	483	Sand, gravel, stones	Forest/Budzisk Nature Reserve	Low
K6	53° 16′ 53.3″ N 23° 22′ 27.2″ E	Migówka catchment → Sokołda catchment	n.a.	Rheocrene	Round	500	Sand, gravel, stones	Forest/Budzisk Nature Reserve	Low
K7	53° 14′ 05.1″ N 23° 20′ 09.2″ E	Jałówka catchment	2.7	Rheocrene	Round	368	Sand, gravel	Forest/Jałówka Nature Reserve	Low
K8	53° 16′ 41.8″ N 23° 07′ 12.6″ E	Krzemianka catchment → Czarna catchment	10.9	Rheocrene	Round	561	Sand, gravel	Forest/Krzemianka Nature Reserve	Low
K9	53° 08′ 21.9″ N 23° 33′ 02.1″ E	stream from Sofipol catchment	15.2	Rheocrene	Round	310	Sand, gravel	Grassland/alder forest	Low
K10	53° 05′ 39.0″ N 23° 30′ 21.9″ E	Świniobródka catchment → Płoska catchment	4.5	Rheocrene	Round	405	Sand, gravel	Forest	Low
K11	53° 11′ 13.3″ N 23° 18′ 53.3″ E	Pilnica catchment	4.9	Rheocrene	Elongated	171	Sand, gravel	Forest/Krasne Nature Reserve	Low

The study of vascular plants and bryophytes was carried out in August 2015, in the middle of the growing season. All plants were determined to the species level, and the quantity and sociability of individual plant species were determined using the Braun-Blanquet scale (Braun-Blanquet 1932). Taxonomic nomenclature of vascular plants was adopted based on Mirek et al. (2002), mosses according to Ochyra et al. (2003), and liverworts after Szweykowski (2006). Plant name abbreviations have been created from six-letter codes, for example *Lemna minor*—*Lemmin.*

Hydrochemical studies were conducted at the same time as the identification of plant species. Water temperature, pH, electrolytic conductivity (EC), oxygen concentration, and water saturation with oxygen were determined with a HachLange multiparameter probe (HQ40). Chemical water analyses were carried out in accordance with ISO standards, by means of methods described previously (APHA 1992). The following analyses were performed: calcium (Ca^2+^) by means of titration with disodium EDTA against calcite, sulfate (SO_4_^2−^) by the nephelometric method with barium chloride, chloride (Cl^−^) by a method with mercury thiocyanate, ammonium nitrogen (NH_4_^+^-N) by the indophenol method, and nitrate nitrogen (NO_3_^−^-N) by the reduction method with *N*-(1-naphthyl) ethylenediamine. The remaining water chemical parameters were determined by the spectrophotometric method with SpectraMax M2 with the application of procedures and reagents by Riedel-de Haen. Soluble reactive phosphorus (SRP) was determined by the molybdenum method after filtering through a “GF/C” filter, total phosphorus (TP) by the molybdenum method after prior acidification, and mineralization with UV light. The concentration of dissolved organic carbon (DOC) was determined with a Shimadzu TOC-L analyzer according to the method described by Zieliński and Górniak (1999).

### Data analysis

Species richness parameters were determined for each community type, separately for bryophytes and vascular plants. We measured species diversity as calculated according to Shannon’s formula (Shannon and Weaver 1963):

$$ {H}^{\prime }=-\sum \limits_{i=1}^R{p}_i\mathit{\ln}\ {p}_i $$

*p*_*i*_: proportion of total cover in the stream represented by the species

The index of floral originality (IFO) was determined by using the number of rare species and/or elusive species (Ejsmont-Karabin 1995):

$$ \mathrm{IFO}=\frac{\sum_{i=1}^k\frac{1}{m_i}}{s}\times 100\% $$

IFO: index of floral originality
*m*: the number of trials (number of spring niches) in which the species occurs
*s*: the number of species per sample (spring niche)

The statistical analyses were conducted with the application of XLSTAT 2016.1. and PS IMAGO 4.0. Spearman correlation was used to find relationships between bryophyte and vascular plant species richness within each spring niche. Principal component analysis (PCA) (Ouyang 2005) and principal factor analysis (PFA) were used to determine the number of principal components or factors to be retained for further study. A commonly used criterion for the number of factors to rotate is the eigenvalues-greater-than-one rule proposed by Kaiser (1960). An eigenvalue less than one implies that the scores on the component would have negative reliability, and there are as many reliable factors as there are eigenvalues greater than one (Cliff 1988).

Canonical correspondence analysis (CCA) was used for relating the composition of macrophytes to environmental variables. CCAs are not always appropriate for composition data, but community ecologists have repeatedly argued that the Euclidean distance is inappropriate for raw species abundance data involving null abundances (Legendre and Legendre 1998). Moreover, CCA can be used in short and long gradients of species data (Legendre and Gallagher 2001). Two-way MANOVA was used for testing differences among springs, with spring area as a covariate. The significance of differences of mean values of biodiversity indicators between springs located in Białystok city and in KFLP was assessed using Statistica 12.5 software, separately for vascular plants and bryophytes. Prior to the analysis, two assumptions were verified: the normality of data distribution and variance homogeneity. To assess the normality of distribution of variables in groups, the Shapiro-Wilk test and analysis of categorized normality charts were used. Based on the test probabilities *p* ≤ 0.05, no grounds were found to reject the assumptions on normality of distributions of all analyzed parameters. The assumption of variance homogeneity was verified applying Levene’s test and the Brown-Forsythe test. Results obtained from Levene’s test indicate failure to meet the variance homogeneity assumption (*p* < 0.05) for all biodiversity indicators (except from IFO) calculated for vascular plants. Because the assumption of normal data distribution was met and the assumption of homogeneity of variance was not, Welch’s *t* test for independent samples was applied.

## Results

### Morphology of spring niches

The average area of spring niche in the KFLP was 338 m^2^ (max = 561 m^2^, min = 160 m^2^), whereas in the urban area, it was 25% lower (max = 500 m^2^, min = 28 m^2^). Over 75% of studied spring niches were characterized by sandy-gravel bottom (Table 1). The bottoms of other niches were filled with sandy formations and, rarely, gravel with a thick detritus layer. In terms of hydrobiology, the majority of outflows were characterized as rheocrene springs, descensive outflows in which groundwater leaks and is filtered from the bottom and niche edges and then flows as a concentrated stream in accordance with the natural land decline. Spring no. B5, located in the Jaroszówka stream valley, was defined as an ascensive outflow, displaying the pulsation of water under low hydrostatic pressure with a direction opposite from gravity. Ascensive outflows are very rare in lowland areas. The study involved two limnocrenes: one from the forested area in KFLP and one artificial limnocrene from the urban environment in Białystok. Due to their location and relationship to the area morphology, most springs were classified as valley-edge, and only a few occurred in the river valleys (Table 1). The anthropogenic impact on spring niches was determined by the distance from built-up areas and communication routes.

### Chemical characteristic of spring water

Tested water from springs was characterized by slight alkalinity (pH = 7.8–8.2). Statistically significant differences between water chemistry of urban and natural spring niche location were identified for pH, EC, the concentrations of calcium, total iron, ammonium, and nitrate ions; and the concentration of DOC (Table 2). A correlation matrix of chemical variables is presented in Table 3.

**Table 2:** Summary statistics for spring water chemistry (significant differences calculated with the Mann-Whitney *U* test, *p* < 0.05)

Stands		Springs B1-B5				Springs K1-K11				Statistical difference (*p* value)
Parameters	Unit	Average	Median	Min	Max	Average	Median	Min	Max	
pH		8.2	7.9	7.6	9.0	7.8	7.8	7.2	8.6	*0.002*
EC	μS cm^−1^	589	580	364	897	394	395	149	588	*0.036*
Ca^2+^	mg dm^−3^	101.3	103.2	73.3	139.4	73.6	72.9	25.5	98.8	*0.047*
Cl^−^	mg dm^−3^	18.3	20.1	7.7	29.9	10.1	9.1	3.6	41.5	0.396
SO_4_^2−^	mg dm^−3^	85.0	88.6	39.3	117.9	53.0	49.2	14.1	87.0	0.062
Total Fe	mg dm^−3^	1.54	1.25	0.57	3.55	0.84	0.60	0.13	4.00	*0.002*
DOC	mg dm^−3^	4.5	3.5	2.6	9.5	5.6	3.8	1.0	22.1	*0.036*
NO_3_^−^-N	μg dm^−3^	310	120	25	1334	617	203	66	4576	*0.002*
NH_4_^+^-N	μg dm^−3^	129	129	63	233	167	174	32	345	*0.002*
Total P	μg dm^−3^	73	60	37	164	83	68	28	167	0.234

Values in italic are significant at 0.05 level

**Table 3:** Correlation matrix (Pearson (*n*)) of the chemical parameters of spring water

Variables	pH	EC	Ca^2+^	Cl^−^	SO_4_^2−^	Total Fe	DOC	NO_3_^−^-N	NH_4_^+^-N	Total P
pH	1	0.457	− 0.441	0.853	0.827	0.809	− 0.029	− 0.485	− 0.369	0.061
EC	0.457	1	− 0.249	0.428	0.568	0.418	0.010	− 0.495	− 0.043	− 0.344
Ca^2+^	− 0.441	− 0.249	1	− 0.345	− 0.167	− 0.318	0.242	0.000	− 0.181	− 0.041
Cl^−^	0.853	0.428	− 0.345	1	0.750	0.773	0.309	− 0.480	− 0.532	0.096
SO_4_^2−^	0.827	0.568	− 0.167	0.750	1	0.615	0.118	− 0.603	− 0.503	− 0.191
Total Fe	0.809	0.418	− 0.318	0.773	0.615	1	0.014	− 0.377	− 0.302	0.049
DOC	− 0.029	0.010	0.242	0.309	0.118	0.014	1	0.110	− 0.544	− 0.055
NO_3_^−^-N	− 0.485	− 0.495	0.000	− 0.480	− 0.603	− 0.377	0.110	1	0.108	− 0.091
NH_4_^+^-N	− 0.369	− 0.043	− 0.181	− 0.532	− 0.503	− 0.302	− 0.544	0.108	1	0.032
Total P	0.061	− 0.344	− 0.041	0.096	− 0.191	0.049	− 0.055	− 0.091	0.032	1

**Fig. 2:**
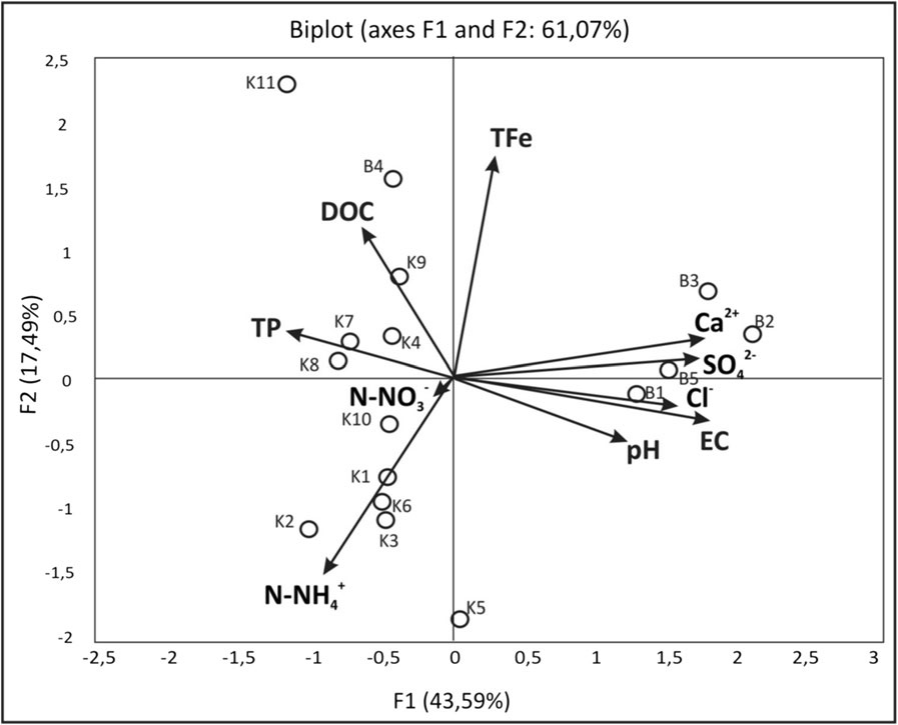
Adjusted biplot scores of chemical variables with sampling sites indicating the measure of fit with the significance level (*p* < 0.05)

PFA results show that the first five principal factors have eigenvalues greater than unity and explain, respectively, 43.6, 17.5, 13.0, 10.0, and 5.8% of the total variances in the original data set. Therefore, the first five factors were used for further analysis. Table 4 shows squared cosines of the variables for the first five factors. The most important water quality parameters that can be used to evaluate variations of the spring water chemistry in NE Poland are the mineral-related parameters (electrolytic conductivity, Ca^2+^, SO_4_^2−^, and Cl^−^). They may be interpreted as representing influences from both natural and anthropogenic inputs. The organic-related parameters (DOC) and nutrients (TP, NO_3_^−^-N) were negatively involved in water quality variations. They may be interpreted as representing influences from natural inputs. Graphical presentation of the relationships based on spring water variables showed ordering of the niches along the water gradients (Fig. 2). Spring niches no. B1, B2, B3, and B5 located in the urban area were characterized by high concentrations of mineral ions, in contrast to spring niches no. K1–K10, based on the concentrations of nutrients and DOC.

**Table 4:** Squared cosines of the chemical parameters of spring water

	F1	F2	F3	F4	F5
pH	*0.858*	0.022	0.031	0.006	0.026
EC	*0.393*	0.059	0.302	0.001	0.130
Ca^2+^	0.121	0.342	0.050	*0.348*	0.041
Cl^−^	*0.847*	0.020	0.045	0.010	0.008
SO_4_^2−^	*0.811*	0.006	0.035	0.011	0.026
Total Fe	*0.680*	0.012	0.035	0.011	0.024
DOC	0.022	*0.695*	0.000	0.041	0.201
NO_3_^−^-N	0.386	0.032	0.015	*0.450*	0.024
NH_4_^+^-N	0.240	*0.558*	0.008	0.004	0.051
Total P	0.002	0.003	*0.776*	0.121	0.055

For each variable, values in italics correspond to the factor for which the squared cosine is the largest

The ordination of samples and variables on the PCA biplot is depicted in Fig. 2; the variability explained by the first axis is 43.59%, while that explained by the second is 17.49%. Samples from the KFLP are shown within the left side of the biplot, while the right side is occupied by samples from the Białystok spring niches. The two axes in the biplots, as well as the positions of the sites, are distorted (Fig. 2). Total phosphorus, calcium ions, and sulfates are positively associated with the distribution of the spring niche samples in Białystok along the horizontal axis. The position of each variable on the biplot is not necessarily identified with the highest values—it only means that the relevant effect of this variable on sample distribution is very important.

### Compositional and structural differences among spring niches flora and species richness

In total, 47 vascular plant and 45 bryophyte species were recorded from 16 spring niches (Tables 5 and 6), including 33 vascular plant and 25 bryophyte species found at more than one site. There were found 20 vascular plants and 24 bryophytes (20 mosses, 4 liverworts) in Białystok and 41 vascular plants and 40 bryophytes (34 mosses, 6 liverworts) in KFLP. The species of bryophytes and vascular plants in two different locations were moderately correlated (*p* = 0.014 (KFLP) and *p* = 0.035 (Białystok) Mann-Whitney *U* test, *p* < 0.05). While many plants were present in both regions, only 26 vascular plant species occurred in < 20% and 17 vascular plant species in < 12% of the spring niches. Four mosses (*Leptodictyum humile*, *Leptodictyum riparium*, *Plagiomnium elatum*, *Pterigynandrum filiforme*) and one liverwort (*Lophocolea heterophylla*) were identified in the urban area and not found in KFLP. In general, bryophyte species observed in the Białystok region are common and resistant to anthropogenic impact (especially *Leptodictyum riparium*).

**Table 5:** Species of vascular plants found in spring niches with relative abundance and sociability (abund., soc.)

Vascular plant species	Spring niches															
	B1	B2	B3	B4	B5	K1	K2	K3	K4	K5	K6	K7	K8	K9	K10	K11
*Athyrium filix-femina*							+ 0.1	+ 0.1	+ 0.1	1.1	1.1	1.1	+ 0.1			1.1
*Berula erecta*			1.1												+ 0.1	
*Bidens tripartita*														+ 0.1		
*Carex riparia*				2.3										1.1	1.1	
*Chrysosplenium alternifolium*		1.1	2.1	1.1								+ 0.1				+ 0.1
*Cardamine amara*	+ 0.1		2.2	1.2												
*Cicuta virosa*			1.1	1.1												
*Cirsium oleraceum*	1.1			3.2		2.2	4.3	2.1	2.2	1.1			1.1		2.2	
*Epilobium ciliatum*										1.1						
*Epilobium palustre*	1.1	1.1	2.1	1.1		1.1	3.2	2.2	2.3	3.2	1.1	2.2	5.4		2.3	2.2
*Equisetum palustre*			1.1	1.1			1.1		+ 0.1		+ 0.1	+ 0.1				
*Eupatorium cannabinum*														3.2	1.1	
*Filipendula ulmaria*						+ 0.1										
*Galium odoratum*													+ 0.1			
*Galium palustre*			2.2			2.2	+ 0.1		2.3		2.1	+ 0.1		+ 0.1	1.1	1.1
*Geranium robertianum*								+ 0.1		2.2		1.1	1.2	+ 0.1		+ 0.1
*Glyceria fluitans*							3.2	3.2	2.2	1.1		3.2			3.2	
*Glyceria maxima*	2.3	1.1		2.2												
*Impatiens noli-tangere*									1.1	1.1			+ 0.1		1.1	
*Iris pseudacorus*			3.3													
*Lemna minor*	1.2			1.2	2.2	1.2		3.3	1.3	1.2	2.2	2.3				2.2
*Lemna trisulca*									1.3			3.3		2.2		4.3
*Lycopus europaeus*						1.1									1.1	
*Lysimachia vulgaris*						+ 0.1										
*Lythrum salicaria*						1.1										
*Mentha aquatica*							2.2	3.3		3.2	1.1			3.3	2.3	
*Mentha arvensis*										1.1	1.1					
*Mycelis muralis*												+ 0.1				
*Myosotis palustris*									1.2			1.1	1.2	+ 0.1	1.1	1.1
Nasturtium officinale*			2.3	+ 0.1				1.2	4.3	3.2	4.3	2.2	3.2			2.2
*Oxalis acetosella*									+ 0.1	1.1	1.1	+ 0.1	+ 0.1			1.1
*Phragmites australis*					4.5	5.5										
*Poa palustris*					2.2											
*Polygonum amphibium*																+ 0.1
*Ranunculus lanuginosus*									+ 0.1	1.1	1.1		+ 0.1		+ 0.1	
*Ranunculus repens*							+ 0.1	1.1				+ 0.1				+ 0.1
*Ribes spicatum*							1.1	1.1								
*Rumex aquaticus*				1.1		1.2	1.1		1.1							
*Rumex hydrolapathum*															1.2	
*Scrophularia alata*													+ 0.1			
*Scrophularia nodosa*																+ 0.1
*Scutellaria golericulata*						+ 0.1	+ 0.1	+ 0.1								
*Solanum dulcamara*							+ 0.1	1.1							1.1	
*Stellaria holostea*													+ 0.1			
*Thelypteris palustris*			2.2	1.2												
*Urtica dioica*			1.1	1.1	1.1	2.3	2.2	2.2	1.3	1.1	3.2	1.2	1.2	1.2	1.2	1.2
*Veronica beccabunga*		1.1	1.1	1.1				2.1	2.2	1.2		1.1	1.1		2.3	2.2

*Protected species

**Table 6:** Bryophyte species found in spring niches and their protection status

Bryophytes species	Spring niches															
	B1	B2	B3	B4	B5	K1	K2	K3	K4	K5	K6	K7	K8	K9	K10	K11
Liverworts																
*Chilloscyphus pallescens*										+						
*Conocephalum conicum*	+									+		+	+			+
*Lophocolea heterophylla*					+											
*Marchantia polymorpha*								+			+	+		+	+	
*Pellia endiviifolia*	+										+	+	+			+
*Pellia neesiana*									+	+					+	
Plagiochila asplenioides*	+								+	+	+		+			+
Mosses																
*Brachythecium mildeanum*									+	+	+		+			
*Brachythecium rivulare*		+		+		+	+	+	+	+		+		+		+
*Brachythecium rutabulum*						+	+									
*Bryum pallens*									+				+			
*Bryum pseudotriquetrum*												+				
Calliergonella cuspidata*														+	+	
Campylopus pyriformis*								+								
Climacium dendroides*													+			
*Cratoneuron filicinum*	+				+				+	+	+	+	+	+	+	+
*Dicranella staphylina*													+			
Dicranum scoparium*											+		+			
*Homomallium incurvatum*										+						
*Hygrohypnum luridum*											+					
*Hypnum cupressiforme*	+				+		+		+	+	+		+			
Hypnum pratense*											+	+	+			
Leptodictyum humile*		+	+													
*Leptodictyum riparium*	+	+	+	+												
*Mnium hornum*							+	+								
Palustriella communata*													+			
*Plagiomnium elatum*				+												
*Plagiomnium ellipticum*								+						+		
*Plagiomnium medium*							+									
*Plagiomnium undulatum*	+	+			+	+	+		+	+	+	+	+		+	+
*Plagiothecium nemorale*												+				
*Plagiothecium ruthei*							+	+								
*Platyhypidium riparioides*											+					
*Pohlia nutans*							+				+					+
*Pohlia wahlenbergii*							+									
Polytrichum commune*							+	+			+		+			+
*Pseudobryum cinclidoides*													+			
*Pterigynandrum filiforme*				+												
*Rhizomnium punctatum*	+			+	+		+			+	+	+	+			+
*Rhytidiadelphus subpin.*													+			
*Sanionia uncinata*										+						
*Schistidium apocarpum*											+					
*Thuidium abietinum*											+					
Thuidium philibertii*										+	+		+			
Thuidium tamariscinum*									+	+		+				+

*Protected species

There have been identified 11 protected bryophytes (*Calliergonella cuspidata*, *Campylopus pyriformis*, *Climacium dendroides*, *Dicranum scoparium*, *Palustriella commutata*, *Plagiochila asplenioides*, *Polytrichum commune*, *Thuidium philibertii*, *T. tamariscinum*) (*Hypnum pratense* and *Leptodictyum humile*)*.* In Białystok, it was found one protected moss (*Leptodictyum humile*), identified in spring niches B2 and B3, and one protected liverwort (*Plagiochila asplenioides*), identified in spring niche B1.

**Fig. 3:**
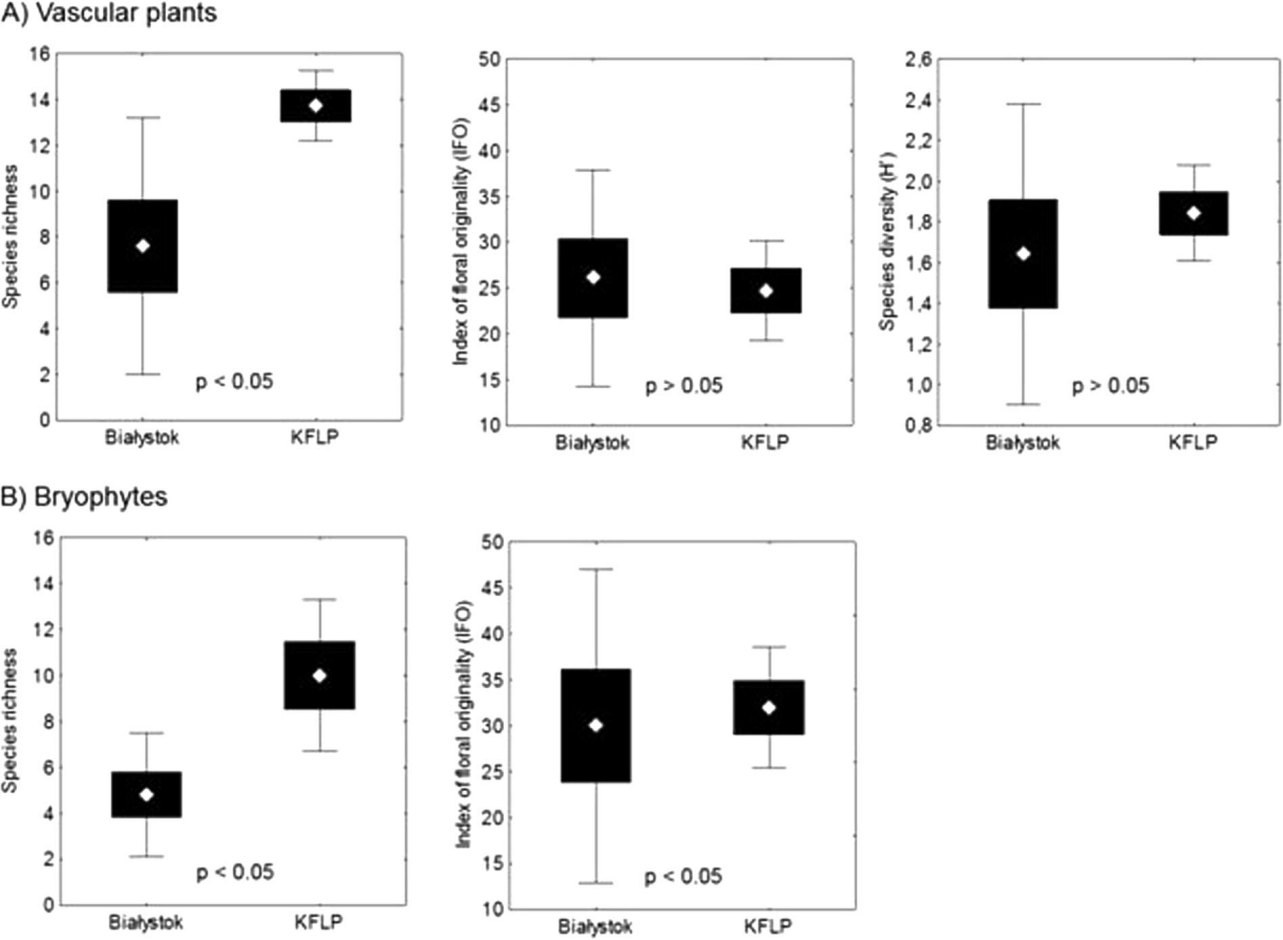
The values of the biodiversity metrics in two locations (mean ± standard error ± 0.95 confidence interval) and results of Welch’s test

The species richness measures for the investigated plant species reflect the diversity of the spring niches in both investigated environments (Table 1). No statistically significant differences were observed for the species diversity index (Fig. 3, Table 7). The greatest floristic diversity was observed in spring niches B4, B2, K5, and K10 and the least in B1, B3, and K8. The index of floral originality indicates the similarity of spring niches in the KFLP in terms of species structure. However, the IFO values in sampling sites B1, B2, and B3 were falsely specified because of very low vascular plant species richness. Spring niches in the KFLP have higher values for the index of bryophyte originality than springs located in Białystok, with the exception of sampling site B4, where the IFO valued 49.2. Using Welch’s *t* test, significant differences in the mean values of biodiversity indicators between springs located in Białystok and in KFLP were assessed separately for vascular plants and bryophytes. On average, species richness of springs located in KFLP showed values two to three times higher than springs in Białystok (Fig. 3).

**Table 7:** Biodiversity indices of vascular plants and bryophytes for each spring niches (statistical significance calculated with the Mann-Whitney *U* test, *p* < 0.05)

	Spring niches																Statistical difference (*p* value)
	B1	B2	B3	B4	B5	K1	K2	K3	K4	K5	K6	K7	K8	K9	K10	K11	
Vascular plants																	
Species richness	5	12	4	13	4	12	13	14	16	15	11	16	14	9	16	15	*0.014*
Species diversity (H′)	1.21	2.28	1.05	2.28	1.00	1.59	1.60	2.16	1.97	2.27	1.82	2.07	1.26	1.36	2.26	1.91	0.533
IFO	19.0	28.2	41.8	21.3	20.1	42.0	20.4	18.6	15.1	22.1	16.3	20.1	32.4	30.7	28.4	25.9	0.910
Bryophytes																	
Species richness	8	4	2	5	5	3	11	7	9	14	17	11	18	5	5	10	*0.035*
IFO	15.7	18.7	37.5	49.	28.7	22.8	40.6	42.9	21.4	36.2	40.9	32.2	45.1	28.0	24.3	17.4	0.692

Values in italic are significant at 0.05 level

Two-way MANOVA shows that there is no statistically significant interaction between water quality and location for species richness with spring area as a covariate (*F* = 51,053, *p* = 0.105; Wilk’s *Λ* = 0.005).

### Effects of environmental variables on individual species

**Fig. 4:**
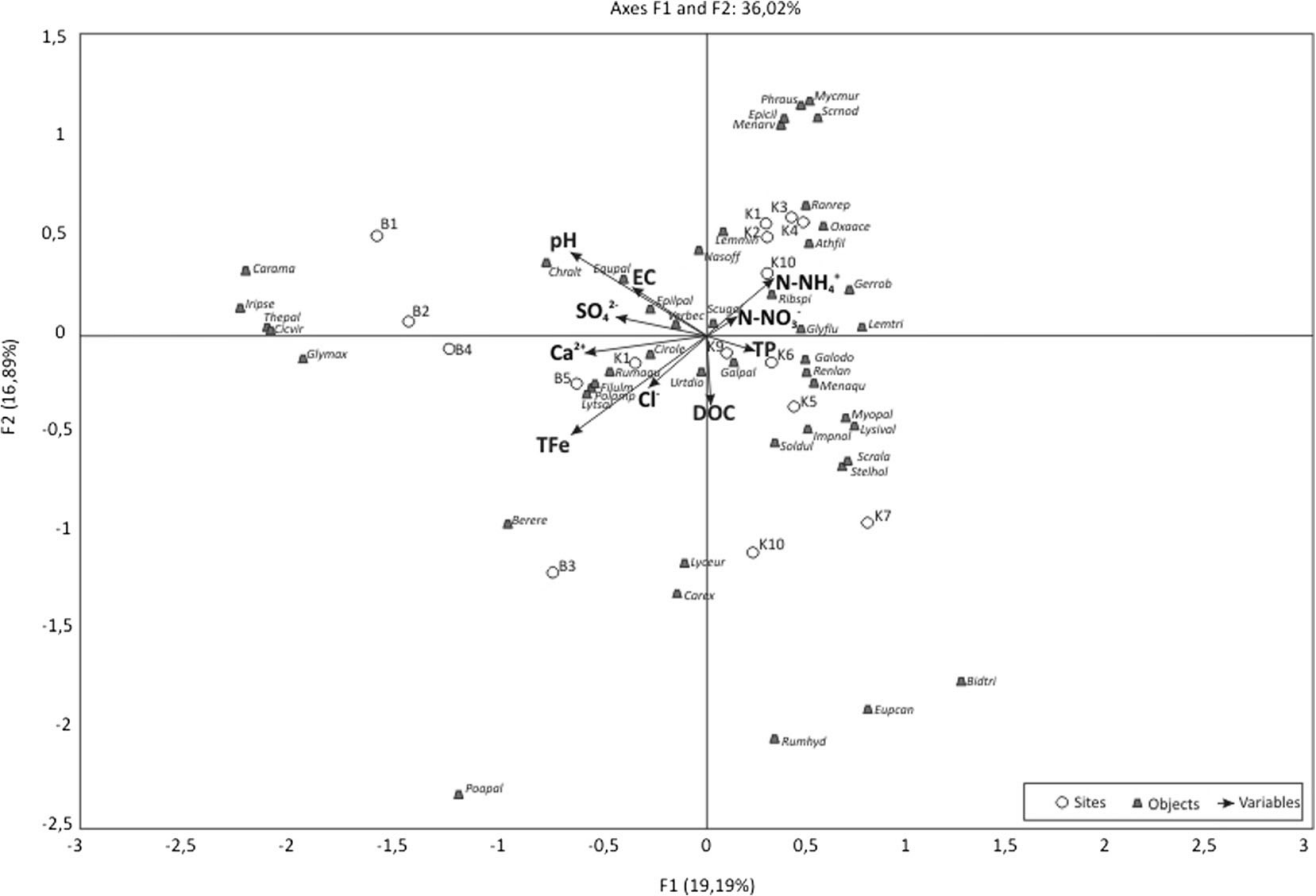
CCA ordination diagram of vascular plants and environmental variables based on 16 sampling sites

**Fig. 5:**
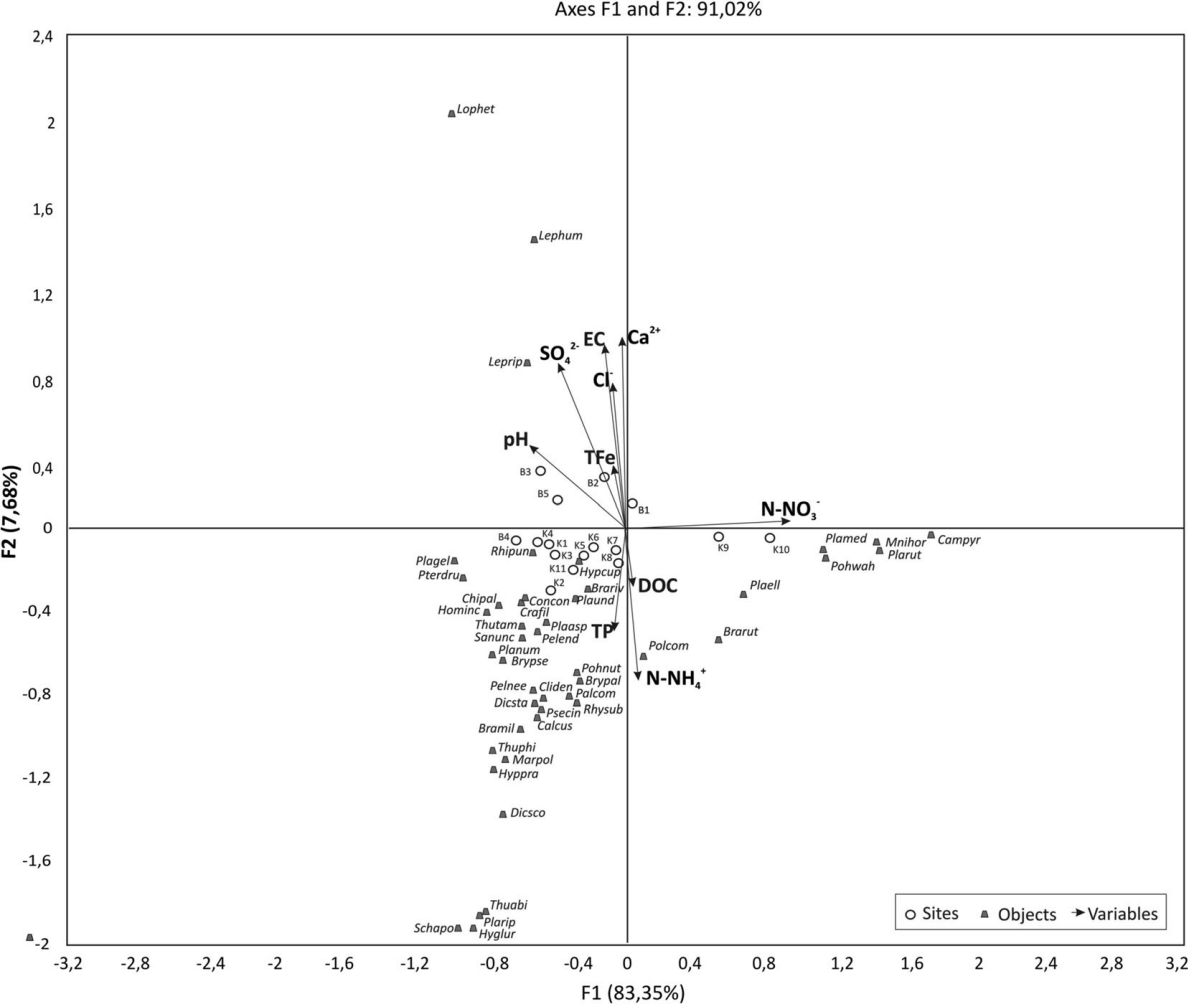
CCA ordination diagram of bryophytes and environmental variables based on 16 sampling sites

The relationship between vascular plants, bryophytes, and environmental variables at the 16 sampling sites was evaluated by CCA and is presented graphically (Figs. 4 and 5). Species data obtained in the study are unimodal along the defined gradients (electrolytic conductivity, NO_3_^−^-N, NH_4_^+^-N, SO_4_^2−^, and Cl). CCA was carried out to assess which of the selected variables characterizing spring niches exerted the greatest impact on the diversity of their floristic composition. The following factors played important roles: the concentration of nutrients, ammonium ions, and DOC. Ordering of vascular plant species based on their quantitative share in niches showed that the existing plants could be grouped according to the habitat gradients—from fertile habitats rich in organic matter to poorly fertile, mineral-rich habitats. The CCA shows that urban spring niches are characterized by the presence of bryophytes related to the anthropogenic environment. The highest number of bryophyte species is associated with those concentrations of selected nutrients and DOC (Fig. 5). In this case, mosses show responses quite different from those of angiosperms, whereas the angiosperm CCA biplot is mostly the same as the water quality PCA.

## Discussion

Our results show that preferences of bryophyte and vascular plant species in the studied spring niches are similar along the main environmental gradients and different in water chemistry. Bryophyte and vascular plant species richness were positively related in all studied sites (*r* = 0.3921; *p* < 0.05), independent of the niche transformation. Vascular plants and bryophytes have similar habitat requirements in lowland springs. This means that similar methods can be used to protect the large-scale richness of those two taxonomic groups in spring niches, as also described in two wetland nature reserves in Estonia (Ingerpuu et al. 2001). However, research in western Norway indicates differences in habitat requirements between vascular plants and bryophytes. For these two taxonomic groups, species richness responds very differently to altitude (Grytnes et al. 2006).

Forty-five bryophyte species representing various ecological groups and growing on a variety of microhabitats were identified in the studied springs: aquatic (e.g., *Brachythecium rivulare*, *Calliergonella cuspidata*, *Platyhypnidium riparioides*), epixylic and epiphytic (growing on logs or stumps of trees, e.g., *Brachythecium rutabulum*, *Lophocolea heterophylla*, *Marchantia polymorpha*), and terrestrial species (growing on soil or humus, e.g., *Polytrichum commune*, *Rhytidiadelphus subpinnatus*, *Thuidium tamariscinum*). Most of the identified bryophyte species are typical of the springs and river valleys of central and eastern Poland (Czarnecka 2009; Czarnecka and Janiec 2007; Mazurek 2008; Mazurek 2006; Grzelak 2011). Similar species were identified by Fudali et al. (2015) during a study on the distribution of bryoflora in the upper valley of Świerszcz stream in Roztocze National Park (75% of common species).

Some evidence was observed for segregation of species according to water chemistry. Several of the environmental factors measured in this study show significant impact on the flora of spring niches (Table 2). The concentration of nutrients was positively correlated with vascular plants in springs in KFLP (Table 7). Most of the springs in KFLP were close to a natural state (Table 1). Furthermore, the degree of naturalness in the catchment does not correlate with species richness (Table 7). Some of spring niches located in urban areas were characterized by higher values of Shannon’s species diversity index than were observed for spring niches in KFLP. The floral originality index was associated with the condition and morphology of spring niches (Table 7). This is particularly evident in two studied limnocrenes: nos. B3 and K4, where CCAs show similarities in the chemical characteristics of water but differences in floral structure resulting from the catchment management (Table 1, Figs. 4 and 5). Spring no. B3, located in an urban area, has a poorer ecological status, with the lowest flora species richness and the lowest Shannon’s species diversity. Bryophytes showed a stronger response than vascular plants to water quality, because bryophytes absorb nutrients only from spring water.

Research by Jekatierynczuk-Rudczyk (2008) indicates that the water quality of springs located in the city is slightly different compared to the hydrochemical background of springs located in the Supraśl river catchment. The surface waters of lowland areas of northeastern Poland, especially in forests and wetlands, are characterized by elevated concentrations of organic matter, which can also be noticed in the springs (Zieliński et al. 2016). Water chemistry of springs is affected not only by flowing groundwater but also by changes in the morphology of the outflow of the spring niche (Chapman et al. 1993). Low temperature reduces the rate of biomass decomposition and favors the accumulation of organic matter (Wołejko 2000). The hydrochemical types of spring water in Bialystok show disturbing symptoms associated with human activity (Jekatierynczuk-Rudczyk 2007, 2008). Our results indicate higher ranges of pH and EC for water from Białystok springs, which probably results from transformations of urban catchment. Our research confirms that the chemical composition and morphological factors play important roles in structuring the floristic composition and diversity of plant communities in springs (Zechmeister and Mucina 1994; Sołtys-Lelek et al. 2014). Furthermore, the species of vascular plants that are characteristic of spring areas, such as *Cardamine amara* and *Chrysosplenium alternifolium*, correspond with urban spring niches, which may be the result of shading. The springs in KFLP were characterized by higher similarity in species composition of vascular plants and bryophytes than in Białystok springs (Tables 5 and 6). The location of springs in forested areas favors the growth of sciophytes.

Many aquatic bryophyte species are used in bio-indicator methods of watercourse assessment (i.e., Haury et al. 2006; Holmes et al. 1999; Meilinger et al. 2005; Szoszkiewicz et al. 2010; Willby et al. 2009). Most of them are typical indicators of low concentrations of nutrients in water (especially orthophosphates; Szoszkiewicz et al. 2010), although a few tolerant species (e.g., *Leptodictyum riparium*, *L. humile*) indicate anthropogenic degradation of watercourses (Ceschin et al. 2012; Vanderpoorten 1999). In the studied springs, concentrations of nutrients were slightly higher in springs located in the KFLP region, but in the vast majority, the nutrient concentrations corresponded to generally accepted criteria for this type of lowland watercourse (Jusik et al. 2015; Pardo et al. 2012; Wallin et al. 2003): total phosphorus < 0.2 mg P dm^−3^, nitrate nitrogen < 1.0 mg N dm^−3^, ammonium nitrogen <0.2 mg N dm^−3^. Probably for this reason, bioindicators of oligotrophy and mesotrophy occurred in many of the studied springs (Table 6): *Brachythecium rivulare*, *Bryum pseudotriquetrum*, *Calliergonella cuspidata*, *Chiloscyphus pallescens*, *Conocephalum conicum*, *Cratoneuron filicinum*, *Hygrohypnum luridum*, *Palustriella commutata*, *Pellia endiviifolia*, *Platyhypnidium riparioides*, and *Schistidium apocarpum*. Meanwhile, springs located in Bialystok city were dominated by two species associated with human activity: *Leptodictyum riparium* and *Leptodictyum humile* (Ceschin et al. 2012).

Compared to 100 taxa identified in the spring niches of the Łódź city (Grzelak 2011), the spring niches of Białystok are poor in vascular plants. However, crenophytes have been recorded (*Cardamine amara*, *Chrysosplenium alternifolium*, *Brachythecium rivulare*, *Leptodictyum humile*, *Plagiochila asplenioides*), indicating that the ecological status of the environment is fairly good. Spring niches located in the Nida river catchment area were characterized by an even lower biodiversity of flora. In 21 springs, only 9 showed the presence of vascular plants, amounting to a range of 5–10 species (Chwalik-Borowiec et al. 2011). The small number of vascular plants was likely related to the strong transformation of outflows (in most cases, wells, chapels, and houses were present). Moreover, spring niches with vascular plants were characterized by a very small surface area of not more than 3 m^2^.

The existence of many valuable species in the spring niches of Bialystok suggests the necessity of their legal protection. In Poland, there are only 134 springs protected by law. The state of knowledge about springs is insufficient, so the increasing anthropogenic pressure and changes in water circulation may affect the disappearance of many natural outflows. The flora of spring niches is a good indicator that can be useful in the planning of preventive measures. Bryophytes are sensitive indicators of environmental changes, and many bryophytes are thus particularly threatened by the degradation of habitats (Juutinen 2011; Vanderpoorten 1999). Studies by Heino et al. (2005) and Kapfer et al. (2012) show that the persistence and stability of bryophyte communities are associated with good conditions of the springs. Floristic diversity research on spring niches in northeastern Poland suggests that the presence or absence of crenophytes is indicative of the ecological status of the groundwater outflow complexes. The presence of species under protection indicates a high degree of naturalness of the environment. The occurrence of *Palustriella commutata* in KFLP is interesting because this species is described as a typically calcicole moss (Bain and Proctor, 1980). However, it also occurs in the crystalline Tatras on siliceous bedrock (Smieja 2014). The geological structure of the Krzemianka Reserve is dominated by carbonate gravels with significant admixture of flint. It is one of the KFLP groundwater outflows where EC is the highest, which confirms the dissolution of carbonates (Jekatierynczuk-Rudczyk 2010).

Anthropogenic activity significantly affects the biodiversity of the flora of lowland springs. Biodiversity in spring niches depends not only on the outflow yield, the geological structure of the saturation zone, and the spring exposure but also on water quality (mainly the concentrations of nutrients, ammonium ions, and DOC). Plant community richness combined with the environmental factors (water quality, geological, morphological, hydrological) can be a good indicator of the ecological status of lowland springs.
